# New insights into the putative role of leucine-rich repeat proteins of *Leptospira interrogans* and their participation in host cell invasion: an *in silico* analysis

**DOI:** 10.3389/fcimb.2024.1492352

**Published:** 2024-12-13

**Authors:** Bruno B. Foltran, João P. Gaspar, Igor R. M. Silva, Henrique M. Pires, Fernanda B. Andrade, Giovanna M. Costa, Julia E. L. Paixao, Luis G. V. Fernandes, Aline F. Teixeira, Ana L. T. O. Nascimento

**Affiliations:** ^1^ Laboratório de Desenvolvimento de Vacinas, Instituto Butantan, São Paulo, Brazil; ^2^ Programa de Pós-Graduação Interunidades em Biotecnologia, Instituto de Ciências Biomédicas, Universidade de São Paulo, São Paulo, Brazil; ^3^ Faculdade de Medicina de Ribeirão Preto, Universidade de São Paulo, Ribeirão Preto, Brazil; ^4^ Infectious Bacterial Disease Research Unit, USDA Agricultural Research Service, National Animal Disease Center, Ames, IA, United States

**Keywords:** *Leptospira*, leptospirosis, LRR proteins, pathogenesis, *in sílico* analysis

## Abstract

Pathogenic *Leptospira* are spirochetes that cause leptospirosis, a worldwide zoonotic disease. Leptospirosis affects humans and animals, with approximately 1 million human infections and 60,000 deaths per year. The diversity of leptospiral strains and serovars allied to the fact that pathogenesis is not yet fully understood, make the development of an effective vaccine against leptospirosis a challenge. Outer membrane and secreted proteins are considered potential antigens since they play a vital role in mediating interactions with host molecules. Several domains or motifs have been reported to participate in the leptospiral infection process. Among them, leucine-rich repeat (LRR) proteins have been highlighted as attractive multipurpose proteins, exhibiting a broad spectrum of ligands and having a putative role in bacterial pathogenesis. Indeed, genome annotation of leptospiral species pointed out that LRR proteins are predominant in pathogenic strains, a feature that corroborates this hypothesis. A few LRR proteins of *L. santarosai*, *L. borgpetersenii* and *L. interrogans* have been studied and their possible role in virulence was proposed. Yet, a mechanistic and broad investigation of LRR proteins was not fully performed. In this review, a comprehensive *in silico* analysis of 21 LRR proteins of *L. interrogans* was performed in relation to structure, function, dynamics and virulent potential that will contribute to understanding the key role of these domains in the underlying mechanisms of leptospiral infection.

## 1 Introduction

The genus *Leptospira* comprises pathogenic and saprophytic species. Pathogenic *Leptospira* spp. are the etiological agents of leptospirosis, a widespread zoonosis, while saprophytic bacteria are free-living environmental organisms. To date, classification based on whole genome sequencing has identified more than 68 species of *Leptospira* described (Vincent et al., 2019; Fernandes et al., 2022), within pathogens (P) and saprophytes (S). These clades are further subdivided into subclades. The subclade P1 is divided in P1+, which includes high virulence pathogens and P1- composed by low-virulence pathogens. Species belong to P1+ group are isolated of mammals and responsible to cause infections in humans and animals. The subclade P2 includes those formerly described as the intermediate and similar the P1- group, being mostly of the species environmental isolates. The subclades S1 and S2 include saprophyte species, where the latter constitutes a new subclade defined after the isolation of new saprophytic species from environmental samples (Vincent et al., 2019; Giraud-Gatineau et al., 2024). Lipopolysaccharide (LPS) structural heterogeneity is the basis for the degree of antigenic variation observed among more than 250 identified serovars. Infection in humans occurs through direct contact with wild or domestic animals’ urine or indirectly by exposure to contaminated soil or water (Adler and de la Peña Moctezuma, 2010).

The initial phase of leptospirosis is characterized by nonspecific flu-like symptoms such as fever, chills, headache and myalgia. The disease can evolve to a severe condition known as Weil’s syndrome, corresponding to 5 – 15% of the reported cases (Bharti et al., 2003), and to leptospirosis pulmonary hemorrhage syndrome (LPHS), which is another severe manifestation of the disease that has been globally reported (McBride et al., 2005)

Although numerous efforts have been made to understand the pathogenic mechanisms involved during leptospiral infection, pathways associated with pathogenesis are not yet fully elucidated. To mitigate disease burden, the development of effective vaccines and diagnostic tests are critical. Outer membrane and secreted proteins are considered potential antigens since they can play a pivotal role in mediating interactions with glycosaminoglycans (GAGs), extracellular matrix (ECM) components, serum components and cell receptors such as cadherins and integrins, and they are readily accessible to host immune defenses. Thus, their characterization is an interesting strategy for elucidating the pathogenesis mechanism of *Leptospira* spp.

Another interesting way to narrow down the identification of proteins involved in bacterial pathogenesis is through the presence of domains and motifs in their structure, especially those that occur mostly in pathogens. Proteins comprising LRR motifs are very interesting, ubiquitous proteins that are present in procaryotes and eukaryotes, including humans (Ng et al., 2011). LRR motifs comprise 20 to 29 amino acid residues having a high proportion of leucine residues (Matsushima et al., 2000; Dolan et al., 2007), and they are found in versatile proteins with diverse functions and cellular locations. It has been reported that LRRs form a flexible framework that can adapt a diversity of interactions, permitting an association of a broad group of ligands (Bell et al., 2003; Kedzierski et al., 2004). Several proteins having these motifs were found to be associated with innate immunity, such as in PAMPs (pathogen associated molecular patterns), and in Toll-like and NOD-like receptors (Inohara et al., 2005; Ng and Xavier, 2011) More recently, a study using mouse macrophages expressing an endogenous NLRP3 mutant lacking the LRR domain showed that removal of this domain reduced NLRP3 inflammasome stimulation in mouse macrophages (Duan et al., 2022). The majority of plant and animal immune receptors have an LRR domain, and various functions are attributed to this motif (Padmanabhan et al., 2009). Beyond innate immunity, they participate in wide-ranging functional processes, such as DNA repair, cell adhesion, signal transduction, development, transcription, and RNA processing (Ng et al., 2011).

From the microbial viewpoint, the cell surface-associated protein (BspA) of the human periodontopathogen *Bacteroides forsythus* encompasses 14 complete repeats of 23 amino acid residues with homology to LRR motifs. This LRR protein is predicted to mediate the binding of bacteria to ECM components and to clotting factors, and it probably has a role in bacterial colonization (Sharma et al., 1998). One of the most studied bacterial proteins containing LRR domains is represented by a family of 9 proteins called internalins of *Listeria monocytogenes*, a food-borne bacterium that causes gastroenteritis, meningitis, or abortion. Members of this family are associated with mammalian host cell invasion (Marino et al., 2000.) through interaction with different host receptors to induce infection of human cells, involving complex mechanisms that ultimately result in disease (Ireton et al., 2021). Other pathogens having virulence factors containing LRR domains are *Yersinia pestis* (YopM) (Leung et al., 1990), *Salmonella* Typhimurium (SspH1, SspH2 and SlrP) (Miao et al., 1999), and *Shigella flexneri* (IpaH) (Hartman et al., 1990; Fernandez-Prada et al., 2000)

In *Treponema pallidum*, the syphilis spirochete, an LRR protein (TpLRR) was identified, and although its function remains to be determined, the presence of LRR domains participating in protein-protein and/or protein-lipid interactions could facilitate the associations between molecules of the *T. pallidum* cell envelope (Shevchenko et al., 1997). LrrA, the cell surface-associated LRR, of *T. denticola*, was reported to play a role in the attachment to and infiltration of human epithelial cells, and in coaggregation with another periodontal pathogen *Tannerella forsythensis*. These properties of LrrA suggest this protein to be an important virulence factor for these oral spirochetes (Ikegami et al., 2004).

Genome annotation of *L. interrogans* identified at least 20 encoding LRR-containing proteins, while *L. borgpetersenii* has at least 5, and the saprophyte *L. biflexa* genome bears only one annotated LRR protein-encoding gene (Picardeau, 2017; Eshghi et al., 2019). In pathogenic *Leptospira* spp., several LRR proteins were studied. In *L. santarosai* serovar Shermani, the crystal structure of LRR20 protein, encoded by the gene LSS_11580, was resolved and the recombinant protein (rLRR20) bound to human epithelial cadherin (E-cadherin) (Hsu et al., 2021). In addition, the recombinant LRR38 encoded by LSS_01692 interacted with fibronectin, collagen IV, and Toll-like receptor 2 (TLR2); furthermore, rLRR38 induced inflammation involving the NF-κB and MAPK signal transduction pathways, suggesting its participation during infection (Hsu et al., 2021). Leptospiral LRR proteins were identified from the genome of *L. borgpetersenii* serogroup Sejroe, the main agent of bovine leptospirosis, and two recombinant proteins, rKU_Sej_LRR_2012M (2012) and rhKU_Sej_LRR_2271 (2271), had their immune protective activities evaluated in a hamster model of leptospirosis. rKU_Sej_LRR_2271 showed promising results, inducing high protective efficacy and tissue clearance after heterologous challenge, and it was suggested to be a potential vaccine candidate against animal leptospirosis (Prapong et al., 2022). The crystal structures of 4 LRR proteins of *L. interrogans* serovar Copenhageni strain Fiocruz L1-130 were solved, LIC_12234, LIC_10831, LIC_11098 and LIC_12759 (Miras et al., 2015), and two of them, rLIC_10831 and rLIC_11098 proteins, were further characterized; rLIC_10831 is a vascular endothelial (VE)- and E-cadherin-binding protein (Eshghi et al., 2019), while rLIC_11098 showed a broader spectrum of interaction with host components (Gaspar et al., 2024).

In this paper, we discuss the *in silico* analysis of 21 LRR proteins of *L. interrogans* in terms of structure, function, dynamics and virulent potential and provide new insights into their participation in pathogenicity mechanisms of pathogenic *Leptospira* spp.

## 2 Methods

### 2.1 *In silico* analysis of operon structure, cellular localization, signal peptide and conserved domains in the LRR proteins

The amino acid sequences of the 21 LRR proteins from *L. interrogans* serovar Copenhageni strain Fiocruz L1-130 (Nascimento et al., 2004; Miras et al., 2015) were obtained from the NCBI database. Protein sequences were analyzed by SMART web server (http://smart.embl-heidelberg.de) (Letunic et al., 2021) to determine the presence of conserved domains. Signal peptide and lipoprotein predictions were assessed by SMART, SignalP v. 5.0 (https://services.healthtech.dtu.dk/services/SignalP-5.0/) and LipoP v. 1.0 (https://services.healthtech.dtu.dk/services/LipoP-1.0/) (Juncker et al., 2003) web servers. PSORTb 3.0 (https://www.psort.org/psortb/) (Yu et al., 2010) and Cello v. 2.5 (http://cello.life.nctu.edu.tw) (Yu et al., 2004) web servers were used to determine cellular localization. The physicochemical parameters predicted molecular mass and theoretical pI were analyzed by the ProtParam tool, available at the website (https://web.expasy.org/protparam/) (Gasteiger et al., 2005). Operon analysis of 21 LRR sequences was performed by using the MicrobesOnline program (http://www.microbesonline.org/operons/gnc267671.html) from the genome of *L*. *interrogans* serovar Copenhageni strain Fiocruz L1-130 and the found data was compared with the results *in vitro* obtained by RNAseq (Zhukova et al., 2017).

### 2.2 Proteomics data analysis

The data of the LRR proteins from *L. interrogans* proteome analysis were also collected. For this purpose, the studies of Malmstrom et al. (2009) and (Sarma et al., 2021) were used. Malmstrom and collaborators used a strategy that combines mass spectrometry-based proteomic methods to determine the number of protein copies per cell, while Sarma et al. (2021) estimated the relative abundance of the proteins in the sample by using the iBAQ (intensity-based absolute quantification) algorithm.

### 2.3 Virulence prediction of LRR proteins and vaccine target prediction

VirulentPred 2.0 (https://bioinfo.icgeb.res.in/virulent2/predict.html) was used to predict bacterial virulence factors. This web server is based on a two-layer cascade support vector machine, responsible for predicting virulent proteins in reliance on a repertoire of experimentally validated virulent protein sequences (Sharma et al., 2023). The VaxiJen v2.0 web server (https://www.ddg-pharmfac.net/vaxijen/VaxiJen/VaxiJen.html) was used to predict possible protective antigens regardless of alignment, allowing the classification of antigens only by the physicochemical properties of the proteins (Doytchinova and Flower, 2007).

### 2.4 Structural analysis

Structural analysis of LRR proteins of *Leptospira* spp. were modelled using CollabFold v15.5 (Mirdita et al., 2022), which is based on the AlphaFold2 protein structure prediction algorithm - AlphaFold2/CollabFold (https://colab.research.google.com/github/sokrypton/ColabFold/blob/main/AlphaFold2.ipynb) (Jumper et al., 2021; Mirdita et al., 2022; Varadi et al., 2024). For comparison, the crystal structures of LIC_10831 (Miras et al., 2015; Eshghi et al., 2019) and the virulent factor InlB (PDB 1D0B) of *Listeria monocytogenes*, were used. The structures were aligned in the PyMOL Molecular Graphics System, Version 2.5.5 Schrödinger, LLC software (https://pymol.org/-page-top) using CE algorithm, and the root mean square deviation was used to classify proteins as homologous (under 1.0), similar (from 1.0 to 3.0) and different (above 3.0). Sequences were aligned using MAFFT algorithm in Jalview software (Waterhouse et al., 2009), for identification of homologous regions, and by the Basic Local Alignment Search Tool (BLAST) web service (https://blast.ncbi.nlm.nih.gov/Blast.cgi) (Altschup et al., 1990), to obtain query cover and percentage identity.

### 2.5 Prediction of molecular, biological and cellular function

Gene Ontology search was performed using Argot 2.5 web server (https://www.medcomp.medicina.unipd.it/Argot2-5/) (Lavezzo et al., 2016), with a threshold of 200, annotating IDs and hits for molecular function, biological process and cellular component.

### 2.6 Conservation analysis of LRR proteins among *Leptospira* spp.

BLAST was used to align the LRR sequences of *L. interrogans* serovar Copenhageni strain Fiocruz L1-130 against different species of *Leptospira* from the subclades P1, P2, S1 and S2. The coverage and identity percentages were used to calculate a conservation value (c-value) among the orthologs of LRR proteins. The c-value was expressed as an index between 0.0 (non-present) and 1.0 (conserved) (Lopes et al., 2019; Nascimento Filho et al., 2024), and was calculated as follows:


$$c\;value=\frac{identical+similar\;amino\;acids}{2}.\;coverage$$

C-values were used to generate the heatmap image, and data analysis was carried out using Seaborn version 0.13.2 (Waskom, 2021), NumPy version 1.24.0 (van der Walt et al., 2011), Panda’s version 2.2.2 (Reback et al., 2020) and Matplotlib version 3.9.0 (Hunter, 2007) packages in Python 3.12.

## 3 Results and discussion

### 3.1 Conservation of LRR proteins among pathogenic, intermediate and saprophytic *Leptospira* species

**Figure 1:**
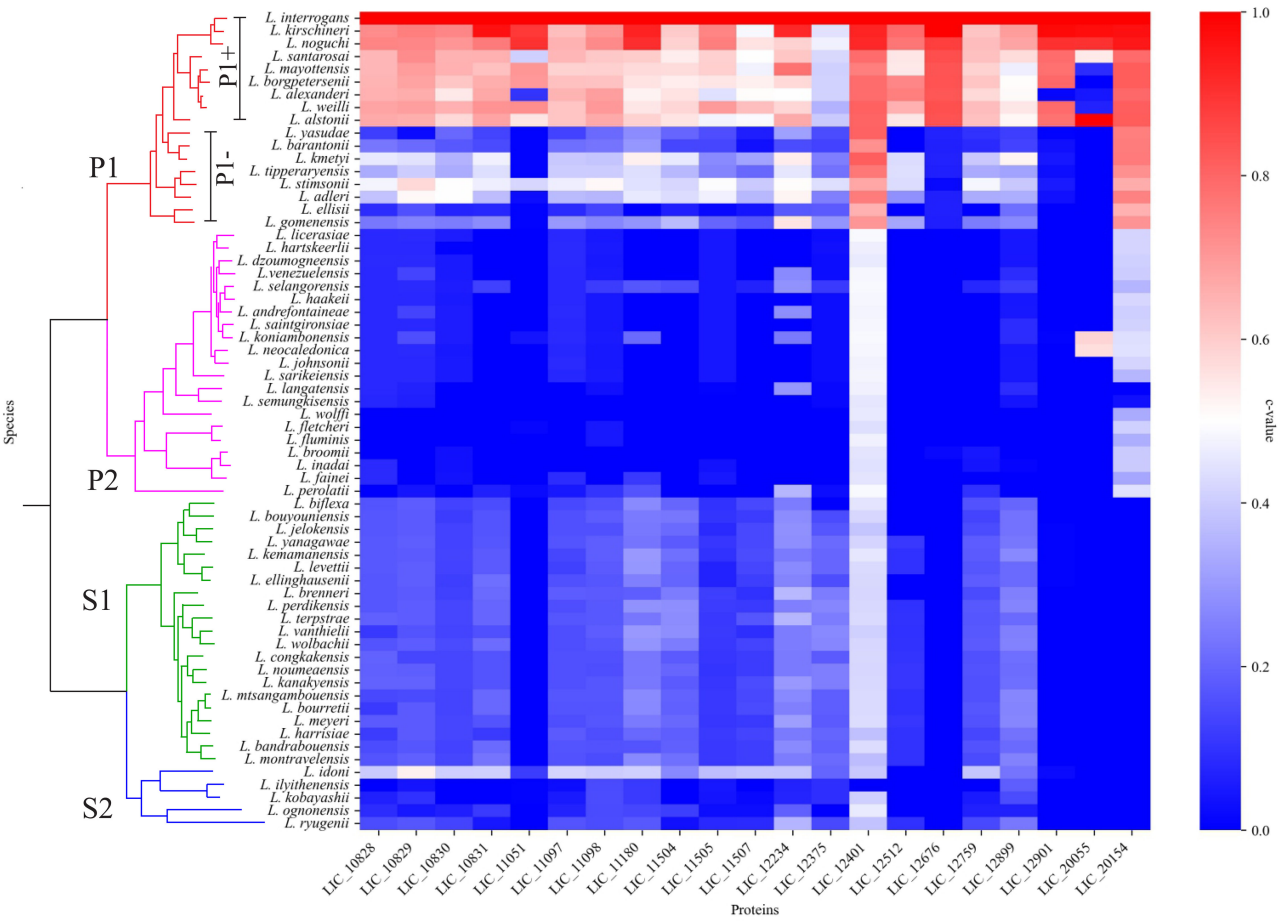
LRR amino acids sequence conservation analysis among the different *Leptospira* spp. Heatmap showing the conservation level among pathogenic (P1+ and P1-), intermediate (P2) and saprophytic (S1 and S2) species of *Leptospira.* Heatmap was elaborated based in the c value among the orthologs of LRR proteins.

The 64 *Leptospira* species classified according to (Vincent et al., 2019) were used to determine the conservation of LRR proteins among the leptospiral pathogenic, intermediate and saprophytic groups. A total of 21 amino acid sequences were identified with the LRR domain encoded by *L. interrogans* strain Fiocruz L1-130 genes, as shown in **Table 1**. Sequences were compared using BLASTp against the predicted protein database of each *Leptospira* species, and a heatmap was created to visualize the conservation level among the different groups. As observed in **Figure 1**, the LRR proteins are well conserved among the pathogenic groups (subclade P1), exhibiting a higher c-value when compared with intermediate (subclade P2) and saprophytic strains (subclade S1 and S2), but only LIC_12401 and LIC_20154 showed identity greater than 70% for all pathogenic species (see **Supplementary Table S1**), except for *L. gomenensis*. Moreover, LIC_20154 is found only in pathogenic (c-value of 0.79) and intermediate species (c-value of 0.39), which can indicate an active role in the infection process. The coverage rate for most intermediate strains was very low, which shows a reduced identity among the sequences. For saprophytic strains, most LRR sequences showed a conservation level below 40% (**Supplementary Table S1**).

**Table 1:** General features of LRR- proteins from *L. interrogans*.

		ProtParam					SMART prediction			
*L. interrogans* Fiocruz L1-130 Gene Locus	TrEMBL	#AA	Mol mass (Da)	pI	PSORTb Localization	Cello Localization	LRR domains	Other domains	Signal P 5.0 Signal peptide (aa)	LipoP 1.0
LIC_10828	Q72U36	378	44104.89	9.12	EC	CYT, OM	12	X	1 to 24 (SPII)	Absent
LIC_10829	Q72U35	402	46312.73	9.23	EC	EC	17	X	Absent	Absent
LIC_10830	Q72U34	521	60165.64	5.85	EC	CYT, EC	17	X	1 to 24 (SPII)	Absent
LIC_10831	Q72U33	377	44197.53	8.94	EC	EC or CYT	13	X	1 to 29 (SPI)	Absent
LIC_11051	Q72TH0	685	78363.38	5.18	EC	OM or EC	7	WGR	Absent	Absent
LIC_11097	Q72TC4	413	48214.35	9.09	EC	CYT or OM	13	X	1 to 38 (SPI)	Absent
LIC_11098	Q72TC3	426	48971.95	8.97	EC	EC or CYT	16	X	1 to 24 (SPII)	1 to 29 (SPI)
LIC_11180	Q72T41	288	33433.13	9.38	EC	CYT	8	X	Absent	Absent
LIC_11504	Q72S80	266	31060.53	9.01	EC	CYT	6	X	Absent	Absent
LIC_11505	Q72S79	572	66278.22	8.82	EC	OM or CYT	17	X	1 to 24 (SPII)	1 to 29 (SPI)
LIC_11507	Q72S77	500	58441.47	9.04	EC	CYT	13	X	1 to 26 (SPI)	1 to 26 (SPI)
LIC_12234	Q72Q78	167	19190.18	5.35	EC	CYT	5	X	Absent	Absent
LIC_12375	Q72PU2	301	35232.28	10.03	EC	CYT or OM	6	X	Absent	Absent
LIC_12401	Q72PR6	217	24581.75	8.65	EC	CYT or OM	3	X	Absent	Absent
LIC_12512	Q72PF9	122	14155.89	9.26	Unknown	CYT	2	X	1 to 23 (SPI)	Absent
LIC_12676	Q72P01	657	76255.87	5.75	CYT	CYT	0	WGR	Absent	Absent
LIC_12759	Q72NS0	423	48762.96	8.82	EC	OM or CYT	14	X	1 to 23 (SPI)	Absent
LIC_12899	Q72ND5	272	31028.95	6.14	EC	CYT or PRP	7	X	1 to 17 (SPII)	Absent
LIC_12901	Q72ND3	1616	184691.17	6.21	EC	OM or EC	6	WGR and DUF4132	Absent	Absent
LIC_20055	Q75FX6	291	33310.26	9.00	CYT	CYT	0	X	Absent	Absent
LIC_20154	Q75FM8	241	28261.02	8.75	CYT	CYT	0	X	1 to 21 (SPII)	Absent

### 3.2 Features of the *L. interrogans* LRR proteins

**Figure 2:**
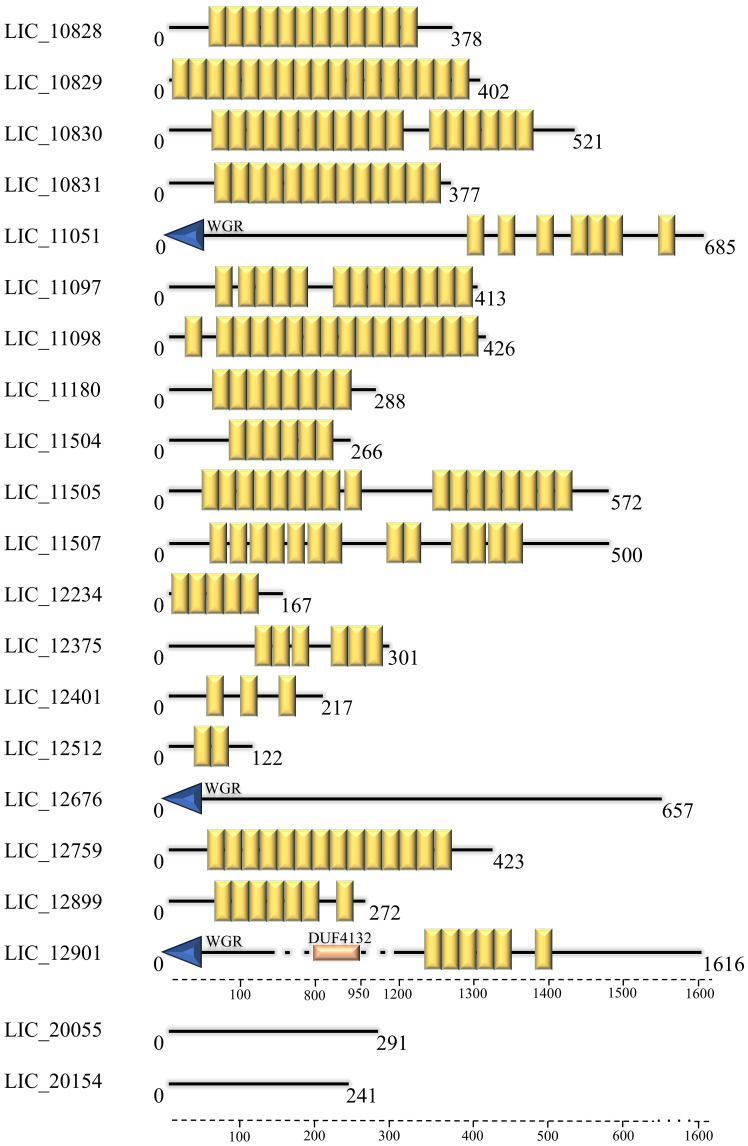
Representative scheme and distribution of LRR domains. Schematic representation of LRR proteins from *L. interrogans* according to SMART webserver (http://smart.embl-heidelberg.de). Each sequence shown in yellow represents a LRR domain; WGR and DUF domains are also demonstrated. Scale is representative of the amino acids sequence.

The amino acid sequences of the 21 putative LRR proteins from *L. interrogans* (Miras et al., 2015) were analyzed *in silico* and their characteristics including number of amino acids, molecular mass, isoelectric point (pI), cellular localization and presence of domains and signal peptide are compiled in **Table 1**. The LRR proteins showed heterogeneous features. Amino acid composition varies from 122 to 1616 amino acids, which results in proteins with a range of molecular mass from 14 to 184 kDa. The majority of LRR proteins exhibits an isoelectric point above pH 7.0, which could suggest the importance of basic amino acid in the protein sequence. The isoelectric point of proteins produced by pathogenic bacteria is a crucial factor that can affect solubility, stability, interaction with host cells, evasion of the immune system, enzymatic activity and biofilm formation, all of which are fundamental aspects for bacterial pathogenesis (Schuurmans Stekhoven et al., 2008). The number of LRR units in each protein is also variable, ranging from 2 to 20 LRR domains, as observed by SMART program. A representative scheme of LRR domains can be visualized in **Figure 2**. In comparison to the prediction performed by Miras et al. (2015), the number of these domains was very similar. However, LIC_12676, LIC_20055 and LIC_20154 were not identified as LRR by SMART, as previously observed (Miras et al., 2015). In addition to LRR domains, the LRR proteins LIC_11051, LIC_12676 and LIC_12901 have a WGR domain and LIC_12901 also possesses a DUF4132 domain, which was previously reported by Sarma et al. (2021). Analysis of signal peptide was performed combining the results generated by SignalP 5.0 and LipoP 1.0. The results produced by the programs were divergent, since LipoP 1.0 was only able to recognize a signal peptide in 3 proteins (LIC_11098, LIC_11505, and LIC_11507), which were all identified as signal peptidase sequence SPI. The results obtained disagree with the SignalP 5.0 analysis, which predicted a SPII for LIC_11098 and LIC_11505. For LIC_11507, the prediction was the same, characterizing it as a SPI protein. Besides LIC_11507, the software did not identify any signal peptide for the LIC_10829, LIC_11051, LIC_11180, LIC_11504 LIC_12234, LIC_12375, LIC_12401, LIC_12676, LIC_12901, and LIC_20055. Comparing our results and the analysis performed by Miras and colleagues (2015), which also used the SignalP 3.0 version, some inconsistencies could be evidenced probably because of the database update of this program. When cellular localization analysis was performed using PSORTb, most LRR proteins were assigned as extracellular, where only LIC_20154, LIC_20055 and LIC_12676 were predicted as cytoplasmic proteins, which corroborates signal peptide analysis, except for LIC_20154. The predictions performed by Cello were diverse, as more than one location was predicted for most of the proteins (see **Table 1**). These distinct results may be due to the different database pool used by the programs. The confirmation of cellular location performed by *in vitro* assays was demonstrated to be extracellular only for LIC_10831, LIC_11098, LIC_11051 and LIC_11505 (Eshghi et al., 2019; Gaspar et al., 2024; Foltran et al., 2024). To verify whether the LRR sequences are in operon, a computational analysis for locating operon structures in *L. interrogans* genome was performed. Of all LRR, the sequences LIC_11097, LIC_11098 together with LIC_11096, the LIC_11180 linked to LIC_11179 and LIC_11181 and LIC_12401 in association to LIC_12400 and LIC_12402 were identified in operon. Interestingly, the genes “LIC_11096, LIC_11097, LIC_11098” and “LIC_11179, LIC_11180, LIC_11181” appear in a mechanism of translational coupling, as observed for the VapBC-1 module of *L. interrogans*, which result in a translation interdependence process (Damiano et al., 2024). Despite their sequential organization, LIC_10828, LIC_10829, LIC_10830, LIC_10831, and LIC_10504 and LIC_10505, these genes are not located in an operon, whereas each sequence is spaced by approximately 200pb, which possibly make the independent transcription process. These in silico analysis agree with the RNAseq analysis by Zhukova et al. (2017).

### 3.3 Proteome analysis of LRR proteins

Experimental data obtained from whole cell and extracellular proteome of *L. interrogans* are highlighted in **Table 2**. Malmstrom et al. (2009) identified all LRR proteins, but only LIC_11051 was quantified with an expected 309 copies/cell. When the copies/spectral count number was calculated, LIC_10828, LIC_11051, LIC_11098 and LIC_12676 were quantified, reaching 27, 132, 75 and 30 copies/spectral count, respectively. In Sarma et al. (2021), LIC_10828, LIC_10831, LIC_11051, LIC_11098, LIC_12676, LIC_12759 and LIC_12901 were identified from Triton X-114 fraction, which contain intracellular outer membrane and secreted proteins. The protein amount was estimated by iBAQ, with the range being from 2.4 to 52.8 iBAQ (**Table 2**). In the secretory fraction, only LIC_11051 and LIC_12901 were detected, reaching 1.2 and 0.1 iBAQ, respectively. Taking into account that the most abundant leptospiral protein, LipL32, is found as having 38,050 copies/cell, according to Malmstrom et al. (2009) and 6388.2 iBAQ in the Triton X-114 fraction (Sarma et al., 2021), and proteins less abundant, such as, FlaB (2,038 copies/cell and 391.5 iBAQ) and DnaK (3,037 copies/cell and 4,128,8 iBAQ), according to Malmstrom et al. (2009) and Sarma et al. (2021), respectively, the data indicate that the LRR proteins occur in low copy numbers in *L. interrogans*.

**Table 2:** Proteome analysis of leptospiral LRR proteins; in grey, proteins characterized by Miras et al. (2015).

		Malmstrom et al. (2009)		Sarma et al. (2021)				
*L. interrogans* Fiocruz L1-130 Gene Locus	TrEMBL	Copies/cell	Copies/spectral counts	Triton X-114 Fractions	Wash	Secretory	Abundance	Pathogenicity
LIC_10828	Q72U36	–	27	10.9	3.6	not found	0.3	P
LIC_10829	Q72U35	–	–	–	–	–	–	–
LIC_10830	Q72U34	–	–	–	–	–	–	–
LIC_10831	Q72U33	–	–	2.4	0.1	not found	0.1	P
LIC_11051	Q72TH0	309	132	25.1	0.4	1.2	0.1	P
LIC_11097	Q72TC4	–	37	–	–	–	–	–
LIC_11098	Q72TC3	–	75	26.2	5.5	not found	0.2	–
LIC_11180	Q72T41	–	–	–	–	–	–	–
LIC_11504	Q72S80	–	–	–	–	–	–	–
LIC_11505	Q72S79	–	–	–	–	–	–	–
LIC_11507	Q72S77	–	–	–	–	–	–	–
LIC_12234	Q72Q78	–	–	–	–	–	–	–
LIC_12375	Q72PU2	–	–	–	–	–	–	–
LIC_12401	Q72PR6	–	24	–	–	–	–	–
LIC_12512	Q72PF9	–	–	–	–	–	–	–
LIC_12676	Q72P01	–	30	14.3	0.1	not found	0	P
LIC_12759	Q72NS0	–	226	52.8	11.3	not found	0.2	P
LIC_12899	Q72ND5	–	–	–	–	–	–	–
LIC_12901	Q72ND3	–	64	15.4	0	0.1	0	P
LIC_20055	Q75FX6	–	–	–	–	–	–	–
LIC_20154	Q75FM8	–	–	–	–	–	–	–
LipL32	Q72SM7	38050	3793	6388.2	895.4	542.7	0.2	P
FlaB	Q72R59	2038	1156	391.5	8.5	7	0	P
DnaK	P61442	3037	3622	4128.8	390.3	39.6	0.1	NP

### 3.4 Antigenicity and virulent factor prediction

Aiming to evaluate the potential of leptospiral LRR proteins as virulent factors and their potential as antigen molecules, the VirulentPred and VaxiJen v2.0 web servers were used. The virulence prediction identified all proteins as a virulent factor, except LIC_10830, assigned as non-virulent (**Table 3**). Although the involvement of proteins containing LRR domains in virulence of pathogens is expected, their roles in *L. interrogans* virulence has not been fully explored. *In vitro* data regarding interactions with cell receptors and host components are available for LIC_10831, LIC_11098, LIC_11051 and LIC_11505, suggesting their potential role in the leptospiral pathogenicity (Eshghi et al., 2019; Gaspar et al., 2024; Foltran et al., 2024). From the data obtained with these proteins, it seems that the interaction with host components correlates with the higher number of LRR domains present in the proteins (>7 LRR domains). When the LRR sequences were analyzed regarding their antigenicity, most of them were identified as non-antigenic. Only LIC_11051, LIC_11504, LIC_12401, LIC_12676, LIC_12901 and LIC_20154 showed scores above 0.4, being classified as possible antigens. Despite this prediction, the recombinant proteins LIC_10831 and LIC_12234 were able to stimulate specific antibodies in rabbit (Eshghi et al., 2019), while LIC_11098 and LIC_11505 were capable of eliciting a robust IgG antibody response in a mouse model (Gaspar et al., 2024; Foltran et al., 2024), denoting the antigenic potential of these LRR proteins. However, the prospective of these proteins as vaccine antigen were not yet evaluated. Only LBJ_2271 from *L. borgepetersenii*, which contain 74.19% identity with LIC_12401 from *L. interrogans*, was evaluated as recombinant vaccine candidate and showed a 75% protective efficacy in immunized hamsters submitted to heterologous challenge with *L. interrogans*. Despite these promising results, sterilizing immunity was not achieved (Prapong et al., 2022).

**Table 3:** Antigenicity and virulent factor prediction.

Server	UniProt	ViulentPred2	VaxiJen v2.0	
Protein	Cod.	Prediction	Prediction	Score
LIC_10828	Q72U36	Virulent	ANTIGEN	0.1129
LIC_10829	Q72U35	Virulent	NON-ANTIGEN	0.1689
LIC_10830	Q72U34	Non-virulent	NON-ANTIGEN	0.1999
LIC_10831	Q72U33	Virulent	NON-ANTIGEN	0.2206
LIC_11051	Q72TH0	Virulent	ANTIGEN	0.4062
LIC_11097	Q72TC4	Virulent	NON-ANTIGEN	0.1817
LIC_11098	Q72TC3	Virulent	NON-ANTIGEN	0.1267
LIC_11180	Q72T41	Virulent	NON-ANTIGEN	0.1961
LIC_11504	Q72S80	Virulent	ANTIGEN	0.4189
LIC_11505	Q72S79	Virulent	NON-ANTIGEN	0.3018
LIC_11507	Q72S77	Virulent	NON-ANTIGEN	0.2944
LIC_12234	Q72Q78	Virulent	NON-ANTIGEN	0.0469
LIC_12375	Q72PU2	Virulent	NON-ANTIGEN	0.2964
LIC_12401	Q72PR6	Virulent	ANTIGEN	0.5094
LIC_12512	Q72PF9	Virulent	NON-ANTIGEN	0.1143
LIC_12676	Q72P01	Virulent	ANTIGEN	0.4507
LIC_12759	Q72NS0	Virulent	NON-ANTIGEN	0.3229
LIC_12899	Q72ND5	Virulent	NON-ANTIGEN	0.2898
LIC_12901	Q72ND3	Virulent	ANTIGEN	0.4569
LIC_20055	Q75FX6	Virulent	NON-ANTIGEN	0.1856
LIC_20154	Q75FM8	Virulent	ANTIGEN	0.4609

### 3.5 Comparative analysis of leptospiral LRR protein structure

**Figure 3:**
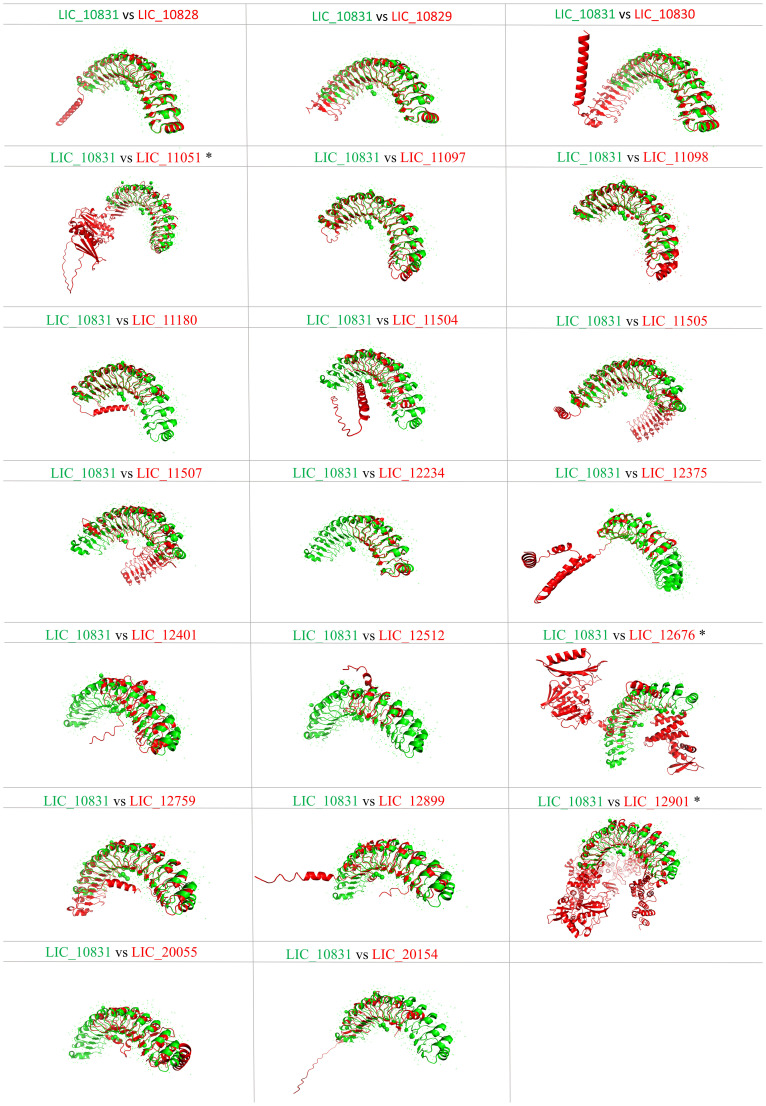
Tertiary structure prediction of *L. interrogans* LRR proteins and spatial alignment with LIC_10831. Amino acid sequences of proteins LIC_10828, LIC_10829, LIC_10830, LIC_11051, LIC_11097, LIC_11098, LIC_11180, LIC_11504, LIC_11507, LIC_11505, LIC_12234, LIC_12375, LIC_12401, LIC_12512, LIC_12676, LIC_12759, LIC_12899, LIC_12901, LIC_20055, LIC_20154 were submitted to the AlphaFold2 program, and the best score was selected. Superposition of LIC_10831 (green) with the other LRR proteins (red) was performed by PyMOL softaware. (*) refers to the presence of WGR domain.

**Figure 4:**
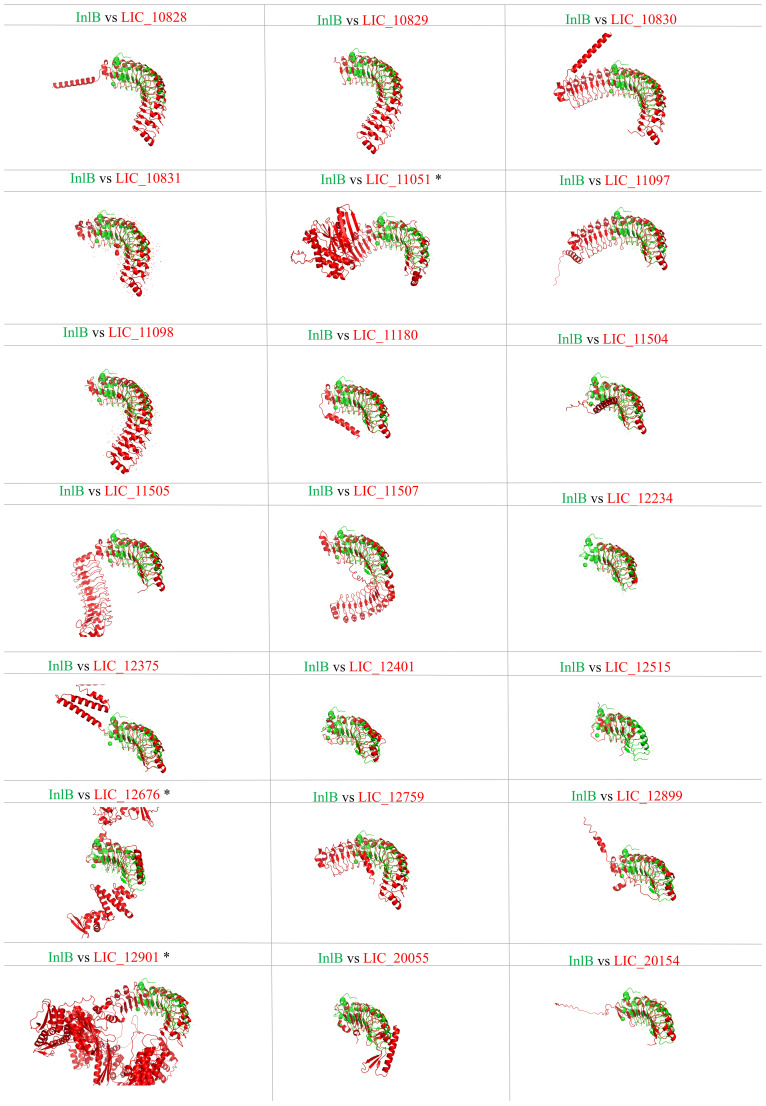
Tertiary structure prediction of *L. interrogans* LRR proteins and spatial alignment with InlB of *L. monocytogenes*. Amino acid sequences of proteins LIC_10828, LIC_10829, LIC_10830, LIC_10831, LIC_11051, LIC_11097, LIC_11098, LIC_11180, LIC_11504, LIC_11507, LIC_11505, LIC_12234, LIC_12375, LIC_12401, LIC_12512, LIC_12676, LIC_12759, LIC_12899, LIC_12901, LIC_20055, LIC_20154. Superposition of InlB (green) with the other LRR proteins (red) was performed by PyMOL softaware. (*) refers to the presence of WGR domain.

Proteins containing LRR domains display curved solenoid structures that may present distinct types of folding being that there are many types of solenoid folds in this protein family. Mainly, the concave side of the LRR domains is marked by a parallel β-sheet, and on the convex side, α-helices, 3_10_ helices, polyproline II helices, β-turns and short β-strands can be found (Bella et al., 2008). It seems that this topology contributes to ligand interaction, and such modules of binding were already characterized in the LRR protein domains (Price et al., 1998; Marino et al., 1999; Kobe and Kajava, 2001; Schubert et al., 2002). Aiming to analyze the leptospiral LRR proteins regarding their structure, we compared the LRR proteins of *L. interrogans* with LIC_10831, whose crystal structure has been solved, containing a distinct binding pocket that may be responsible for interaction with human E-cadherin. Thus, amino acid sequences were submitted to the Alphafold program, and in **Figure 3**, it is possible to observe a spatial coincidence among the structures, even showing a different number of repeats units, denoting that the leptospiral LRRs seems to display a similar curvature radius. For some structures, such as LIC_10828, LIC_10829 and LIC_11097, which share close LRR domain quantities, the overlap was almost identical. In general, the LRR proteins possess a terminal structure that protects the hydrophobic core of the α/β solenoid. It has been observed that the four leptospiral proteins which had their solved structures (LIC_12234, LIC_10831, LIC_11098 and LIC_12759) have the same structural topology, which suggests that not only the stability of the α/β solenoid conformation is maintained but also that specific binding sites are promoted (Miras et al., 2015). Analyzing the N-terminal and C-terminal regions of other leptospiral LRR proteins, we can observe that all proteins share a similar structural topology. However, only LIC_10828, LIC_10829, LIC_10830, LIC_10831, LIC_11097, LIC_11098, LIC_11507 and LIC_12899 overlap identically in the N-terminal region, while for the C-terminal, the only proteins that do not show the same superposition are LIC_12901 and LIC_12676. Both regions contain some conserved residues that are important to maintain the structure, but the C-terminal portion has a greater number of conserved residues among the leptospiral LRRs, mainly arginine and lysine. We have also compared the leptospiral LRR protein structures with internalin B (InlB) from *Listeria monocytogenes*, which is one of the most studied LRR proteins (Marino et al., 1999). Superposition of InlB with the 21 *L. interrogans* LRR proteins shows a similar structural profile (**Figure 4**). Although the repeat unit of the InlB is smaller compared to most leptospiral LRRs, the same curvature radius was observed. LIC_12234, which shares close LRR number to InlB, overlapped almost identically, differing from other LRR proteins showing a similar LRR domain number. As in *Listeria monocytogenes*, InlB belongs to the protein family that is associated with invasion of mammalian host cells (Marino et al., 2000), and it is possible that LIC_12234 may be involved in similar activity (see below).

### 3.6 Analysis of molecular, biological and cellular function by Gene Ontology

**Figure 5:**
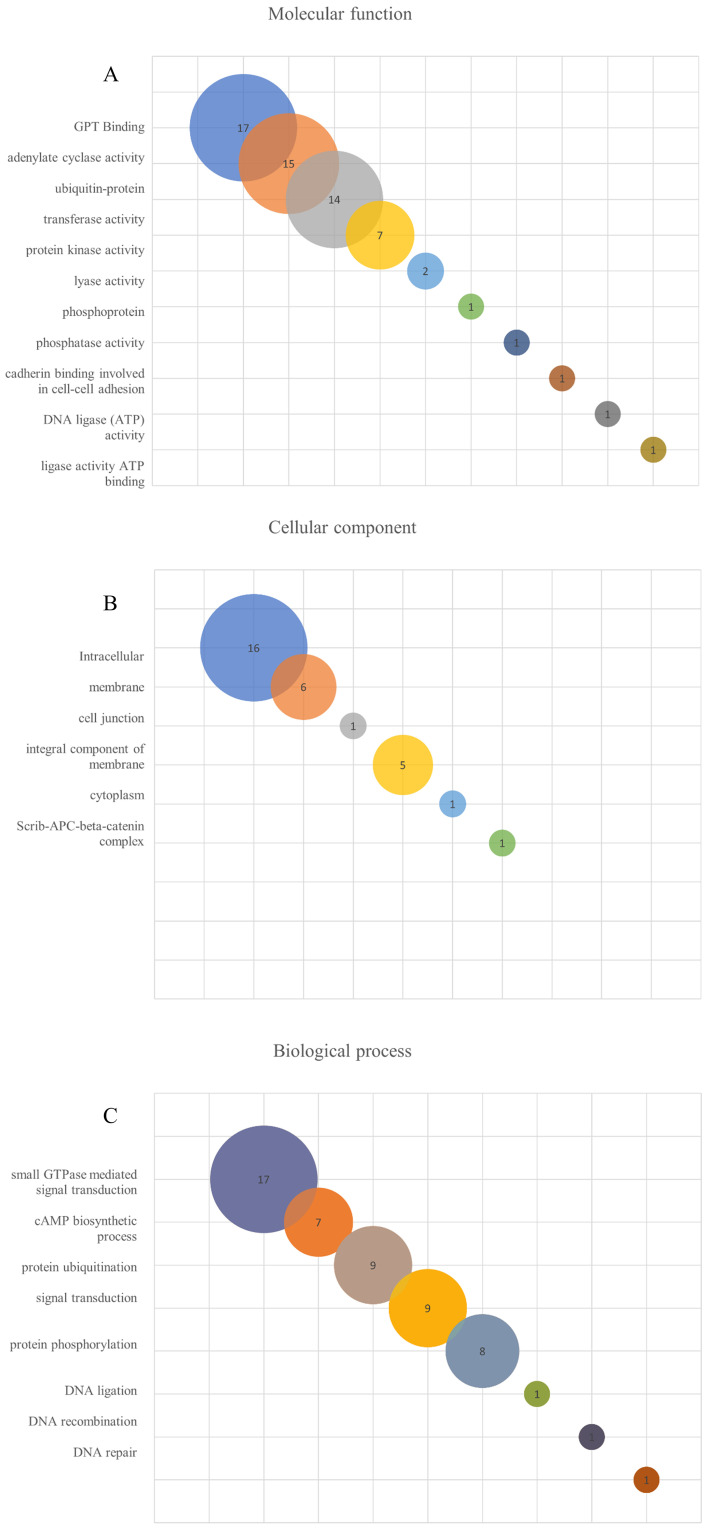
Gene ontology (GO) enrichment analysis of *L. interrogans* LRR proteins. The search was performed using Argot2.5 tool and a threshold of 200 was considered to determine hits for molecular function **(A)**, cellular component **(B)** and biological process **(C)** among the LRR proteins.

Aiming to compare the molecular function, the cellular components and the biological processes involved with the leptospiral LRR proteins, the 21 amino acid sequences were analyzed by the Gene Ontology program. As observed in **Figure 5A** and **Table 4**, analysis related to the molecular function identified 17 proteins associated with GTP binding, which is a regulator protein responsible for cell signaling. That function was also identified in the LRR proteins InlB from *Listeria monocytogenes* and SLR from *S. pyogenes* (**Supplementary Table S2**). Among the GTP-binding protein families in eukaryotes, the proteins of the Rho family play a pivotal role in host cell cytoskeleton dynamics. It participates in signal transduction pathways related to B and T lymphocyte activities, leukocyte migration and phagocytosis and phagocyte degranulation (Aktories, 2011; El Masri and Delon, 2021). The participation of proteins that mimic or covalently modify the host’s GTP-binding protein regulator has already been described for some pathogenic bacteria. For example, included are YopE from *Yersinia* spp. and SopE and SopE2 from *S.* Typhimurium, which act as activators of GTP-binding regulators, and YopT from *Yersinia* spp., which cleaves the host GTPases (Just et al., 2001; Aktories, 2011); 15 of the LRR proteins were identified to have adenylate cyclase activity, which is the enzyme responsible for synthesizing cyclic AMP (cAMP) and 14 were described as ubiquitin-protein transferase activity, which is involved in the regulation of other proteins. In the LRR proteins of other pathogens, this transferase activity related to ubiquitin-protein complex was identified in YopM from *Y. pestis*, SlrP from *S.* Typhimurium and LrrA from *T. denticola* (**Supplementary Table S2**). Seven proteins showing transferase activity were also identified. A low number of LRR proteins were associated with protein kinase activity, lyase activity, phosphoprotein, phosphatase activity, cadherin binding, DNA ligase (ATP) activity, ligase activity and ATP binding. Ligase activity was also observed in YopM from *Y. pestis* and SlrP from *S.* Typhimurium (Wei et al., 2016; Bullones-Bolaños et al., 2024). Additionally, YopM from *Y. pestis*, LrrG from *S. agalactiae*, LrrA from *T. denticola* and TpLRR from *T. pallidum* were associated with ion metallic binding particularly with zinc (Ikegami et al., 2004; Uittenbogaard et al., 2012; Sullivan et al., 2021). Although none of the LRR proteins from *L. interrogans* were associated with ion binding function, it has been described that these proteins are known to bind to divalent cations, such as Zn^2+^ and Ca^2+^ (Miras et al., 2015).

**Table 4:** Gene ontology (GO) analysis of L. interrogans LRR proteins showing the prediction for molecular function, cellular component and biological process among the leptospital LRR proteins.

	*Molecular Function*			*Biological Process*			*Cellular Component*		
*Protein*	ID	Name	Score	ID	Name	Score	ID	Name	Score*
LIC_10828	GO:0005525 GO:0004016 GO:0004842	GTP binding adenylate cyclase activity ubiquitin-protein transferase activity	1.770 619 521	GO:0007264 GO:0006171 GO:0016567 GO:0007165	small GTPase mediated signal transduction cAMP biosynthetic process protein ubiquitination signal transduction	2.801 893 398 258	GO:0005622 GO:0016020	intracellular membrane	1.110 206
LIC_10829	GO:0005525 GO:0004016 GO:0004842 GO:0004672	GTP binding adenylate cyclase activity ubiquitin-protein transferase activity protein kinase activity	1.677 450 403 253	GO:0007264 GO:0007165 GO:0006468	small GTPase mediated signal transduction signal transduction protein phosphorylation	2.241 411 251	GO:0005622 GO:0005634 GO:0005654 GO:0030054 GO:0016021 GO:0016020	intracellular nucleus nucleoplasm cell junction integral component of membrane membrane	835 253 248 230 228 210
LIC_10830	GO:0005525 GO:0004672 GO:0004842 GO:0004016	GTP binding protein kinase activity ubiquitin-protein transferase activity adenylate cyclase activity	1.265 482 381 361	GO:0007264 GO:0006468 GO:0007165	small GTPase mediated signal transduction protein phosphorylation signal transduction	2.411 595 465	GO:0005622 GO:0016021 GO:0016020	intracellular integral component of membrane membrane	969 609 470
LIC_10831	GO:0005525 GO:0004016 GO:0004842 GO:0016829 GO:0004672	GTP binding adenylate cyclase activity ubiquitin-protein transferase activity lyase activity protein kinase activity	1.727 860 391 330 221	GO:0007264 GO:0006171 GO:0006468 GO:0007165 GO:0016567	small GTPase mediated signal transduction cAMP biosynthetic process protein phosphorylation signal transduction protein ubiquitination	2.691 1.194 289 261 232	GO:0005622	intracellular	1.183 -
LIC_11051	GO:0005525 GO:0004016	GTP binding adenylate cyclase activity	751 402	GO:0007264 GO:0006171 GO:0006468	small GTPase mediated signal transduction cAMP biosynthetic process protein phosphorylation	1.045 515 216	GO:0005622	intracellular	544 -
LIC_11097	GO:0005525 GO:0004016 GO:0004842	GTP binding adenylate cyclase activity ubiquitin-protein transferase activity	1.958 505 441	GO:0007264 GO:0016567 GO:0007165	small GTPase mediated signal transduction protein ubiquitination signal transduction	3.066 335 258	GO:0005622 GO:0016021 GO:0016020	intracellular integral component of membrane membrane	1.101 319 221
LIC_11098	GO:0005525 GO:0004016 GO:0004842 GO:0004672	GTP binding adenylate cyclase activity ubiquitin-protein transferase activity protein kinase activity	2.103 570 555 206	GO:0007264 GO:0016567 GO:0007165 GO:0006468	small GTPase mediated signal transduction protein ubiquitination signal transduction protein phosphorylation	3.231 385 359 227	GO:0005622 GO:0016021 GO:0016020	intracellular integral component of membrane membrane	1.271 409 284
LIC_11180	GO:0005525 GO:0004016 GO:0004842	GTP binding adenylate cyclase activity ubiquitin-protein transferase activity	947 523 298	GO:0007264	small GTPase mediated signal transduction	1.117	GO:0005622	Intracellular	262
LIC_11504	GO:0005525 GO:0004016 GO:0004842 GO:0016829	GTP binding adenylate cyclase activity ubiquitin-protein transferase activity lyase activity	1.322 527 268 204	GO:0007264 GO:0006171 GO:0016567	small GTPase mediated signal transduction cAMP biosynthetic process protein ubiquitination	2.172 741 404	GO:0005622	Intracellular	524 -
LIC_11505	GO:0005525 GO:0004672 GO:0004842 GO:0004721	GTP binding protein kinase activity ubiquitin-protein transferase activity phosphoprotein phosphatase activity	867 528 400 228	GO:0007264 GO:0006468 GO:0007165	small GTPase mediated signal transduction protein phosphorylation signal transduction	1.686 594 525	GO:0005622 GO:0016021 GO:0016020 GO:0005737 GO:0005634	intracellular integral component of membrane membrane cytoplasm nucleus	744 448 311 220 210
LIC_11507	GO:0005525 GO:0004016 GO:0004842 GO:0004672	GTP binding adenylate cyclase activity ubiquitin-protein transferase activity protein kinase activity	990 346 264 234	GO:0007264 GO:0007165 GO:0006468	small GTPase mediated signal transduction signal transduction protein phosphorylation	1.379 384 273	GO:0005622	Intracellular	754
LIC_12334	GO:0005525 GO:0004016 GO:0004842	GTP binding adenylate cyclase activity ubiquitin-protein transferase activity	1.129 400 331	GO:0007264 GO:0006171 GO:0016567	small GTPase mediated signal transduction cAMP biosynthetic process protein ubiquitination	1.747 537 429	GO:0005622	Intracellular	475
LIC_12375	GO:0005525 GO:0004016 GO:0004842	GTP binding adenylate cyclase activity ubiquitin-protein transferase activity	1.037 474 251	GO:0007264 GO:0006171 GO:0016567	small GTPase mediated signal transduction cAMP biosynthetic process protein ubiquitination	1.927 707 348	GO:0005622 -	Intracellular	484 -
LIC_12401	GO:0098641	cadherin binding involved in cell-cell adhesion	302	–	–	–	–	–	–
LIC_12512	- -	- -	- -	- -	- -	–	–	–	–
LIC_12676	GO:0003910 GO:0016874 GO:0005524	DNA ligase (ATP) activity ligase activity ATP binding	855 467 339	GO:0006266 GO:0006310 GO:0006281	DNA ligation DNA recombination DNA repair	390 274 262	–	–	–
LIC_12759	GO:0005525 GO:0004842 GO:0004016 GO:0004672	GTP binding ubiquitin-protein transferase activity adenylate cyclase activity protein kinase activity	1.554 509 400 226	GO:0007264 GO:0007165 GO:0016567 GO:0006468	small GTPase mediated signal transduction signal transduction protein ubiquitination protein phosphorylation	2.374 378 308 245	GO:0005622	Intracellular	1.174
LIC_12899	GO:0005525 GO:0004842 GO:0004016	GTP binding ubiquitin-protein transferase activity adenylate cyclase activity	1.275 443 420	GO:0007264 GO:0006171 GO:0016567	small GTPase mediated signal transduction cAMP biosynthetic process protein ubiquitination	2.237 613 526	GO:0005622	Intracellular	613
LIC_12901	GO:0005525 GO:0004016	GTP binding adenylate cyclase activity	882 422	GO:0007264 -	small GTPase mediated signal transduction -	728 -	GO:0034750 GO:0035748 GO:0005622	Scrib-APC-beta-catenin complex myelin sheath abaxonal region intracellular	220 208 205
LIC_20055	–	–	–	–	–	–	–	–	–
LIC_20154	GO:0005525	GTP binding	385	GO:0007264	small GTPase mediated signal transduction	356	–	–	–

* It was considered a threshold of 200.

(**-**) indicates that GO could not associate any data with the protein sequence.

When the cellular components were evaluated, 16 LRR proteins were described as intracellular, 6 of them as membrane proteins and 5 proteins as integral component of the membrane. InlB, YopM, SLR, LrrG, LrrA and TpLRR were membrane associated, while all other LRR proteins were also classified as integral components of the membrane, except YopM. Only 2 LRR proteins have been identified as protein of the nucleus, and one protein as nucleoplasm, cell junction, cytoplasm and scrib-APC-beta-catenin complex (**Figure 5B**; **Table 4**). In relation to biological process, 17 proteins were associated with small GTPase-mediated signal transduction. That function was also observed for InlB from *Listeria monocytogenes* and SLR from *S. pyogenes.* A total of 7 proteins seemed to be involved with cAMP biosynthetic processes, while 9 proteins were related to the protein ubiquitination process. This ubiquitination process seems to occur for the proteins YopM, SlrP and LrrA (Ikegami et al., 2004; Wei et al., 2016; Bullones-Bolaños et al., 2024). Ubiquitination is a reversible post-translational process, usually associated with protein-protein interactions, innate immune signaling, proteasome regulation, cell autophagy and xenophagy (Ashida et al., 2014; Vozandychova et al., 2021). To survive in the host cell, some bacteria have to subvert the host cell machinery. For example, it has been shown that *S. Typhimurium* produces 2 effector proteins, SseL and AvrA, that have deubiquitinating function and are responsible for inhibiting the NF-κβ signaling pathway, which is related to the expression of proinflammatory cytokine genes. Also, *S. pyogenes* produces an effector protein, SpeB, which is a cysteine protease that degrades components of the ubiquitination process, thus avoiding bacterial ubiquitination and host cell autophagy, and *Listeria monocytogenes* possess InlK, that has the ability to bind a host cytoplasmatic protein, avoiding ubiquitination and consequently the xenophagy process (Vozandychova et al., 2021). Nine proteins were identified in signal transduction and 8 in the protein phosphorylation processes. Only one LRR protein out of 21 analyzed sequences were identified as DNA ligand or involved in the recombination and repair processes (**Figure 5C**; **Table 4**).

## 4 Concluding remarks

LRRs are versatile binding domains present in a diversity of proteins that can accommodate a vast type of ligands. Because LRRs are mainly involved in protein-protein interaction, it can be inferred that they are associated with host-pathogen interactions and are used by either the host or pathogen. The comprehensive analysis focused on 21 LRR proteins of *L. interrogans* indicates that they have the potential to have a diverse role in bacteria, which can be extended to other pathogenic *Leptospira* spp. by their orthologous proteins. The results suggest that they could be important for cellular invasion and bacterial survival by altering the host cells’ signaling pathways, suggesting a potential role in infection. Taken together, the data indicate that leptospiral LRR proteins warrant further studies to elucidate their participation in pathophysiological mechanisms, including the selection of targets for mutagenesis in pathogenic *Leptospira*, as a proof of concept of LRR proteins’ role in virulence.
