# Supplementary material for: New insights into the putative role of leucine-rich repeat proteins of Leptospira interrogans and their participation in host cell invasion: an in silico analysis
Source: Front Cell Infect Microbiol. 2024 Dec 13;14:1492352. doi: 10.3389/fcimb.2024.1492352 (PMC11674859; doi:10.3389/fcimb.2024.1492352)
Supplement: Supplementary file 1 [file DataSheet1.pdf]

Table S1: Amino acid sequence identities of LRRs from *Leptospira* spp. Similarity is shown in yellow above 70%, in blue between 40% and 70% and in green below 40%

Proteins	%	LIC10828	LIC10829	LIC10830	LIC10831	LIC11051	LIC11097	LIC11098	LIC11180	LIC11504	LIC11507	LIC11505	LIC12234	LIC12375	LIC12401	LIC12512	LIC12676	LIC12759	LIC12899	LIC12901	LIC20055	LIC20154
L. interrogans	C<br>I	100<br>100	100<br>100	100<br>100	100<br>100	100<br>100	100<br>100	100<br>100	100<br>100	100<br>100	100<br>100	100<br>100	100<br>100	100<br>100	100<br>100	100<br>100	100<br>100	100<br>100	100<br>100	100<br>100	100<br>100	100<br>100
L. kirschineri	C<br>I	100<br>67	100<br>65	100<br>65	100<br>84	100<br>85	95<br>62	100<br>65	100<br>52	95<br>91	90<br>50	99<br>71	100<br>90	81<br>47	100<br>90	100<br>76	100<br>99.39	100<br>57	90<br>68	100<br>98	100<br>97	100<br>94
L. noguchi	C<br>I	100<br>68	100<br>57	100<br>57	100<br>66	100<br>86	98<br>57	100<br>67	98<br>91	95<br>91	95<br>53	99<br>68	98<br>55	81<br>63	100<br>89	100<br>72	100<br>83.87	100<br>58	97<br>52	99<br>88.50	100<br>86	100<br>93
L. santarosai	C<br>I	99<br>58	99<br>58	99<br>58	97<br>62	91<br>39	98<br>56	94<br>59	97<br>52	98<br>52	94<br>46	98<br>52	98<br>57	79<br>60	100<br>73	91<br>54	100<br>79	96<br>48	94<br>53	99<br>73	99<br>46	99<br>71
L. mayottensis	C<br>I	99<br>57	99<br>58	99<br>58	96<br>56	100<br>62	95<br>57	95<br>57	100<br>51	99<br>51	92<br>46	98<br>51	100<br>70	80<br>60	97<br>75	97<br>67	100<br>78	96<br>47	87<br>52	99<br>72	46<br>30	98<br>78
L. borgpetersenii	C<br>I	98<br>56	99<br>60	99<br>60	98<br>60	100<br>61	96<br>55	96<br>56	97<br>56	98<br>56	98<br>45	98<br>48	98<br>54	78<br>61	100<br>74	100<br>65	100<br>78	98<br>53	90<br>48	100<br>74	-	98<br>79
L. alexanderi	C<br>I	98<br>53	100<br>62	100<br>62	100<br>53	46<br>35	97<br>55.	100<br>62	100<br>62	100<br>62	99<br>43	96<br>50	89<br>58	81<br>60	100<br>74	98<br>47	100<br>78	96<br>54	95<br>49	18<br>37	23<br>84	98<br>76
L. weilli	C<br>I	100<br>61	99<br>50	99<br>50	100<br>61	100<br>65	95<br>61	99<br>60	100<br>50	100<br>51	98<br>48	99<br>66	100<br>79	81<br>45	100<br>76	93<br>69	100<br>79	98<br>55	93<br>52	100<br>72	39<br>36	99<br>77
L. alstonii	C<br>I	100<br>60	99<br>58	99<br>58	100<br>60	85<br>68	95<br>56	98<br>56	99<br>56	99<br>56	98<br>42	99<br>51	100<br>55	81<br>48	100<br>77	95<br>46	100<br>78	100<br>52	95<br>47	99<br>73	-	98<br>80
L. dzianensis/ L. yasudae	C<br>I	45<br>35	92<br>33	92<br>33	55<br>30	9<br>77	69<br>30	84<br>30	80<br>30	75<br>30	36<br>34	85<br>31	85<br>35	60<br>32	100<br>76	-	36<br>37	42<br>28	56<br>34	12<br>31	-	96<br>77
L. barantonii	C<br>I	85<br>38	89<br>39	90<br>32	70<br>42	9<br>77	82<br>29	90<br>30	91<br>30	73<br>30	22<br>40	60<br>28	54<br>42	56<br>36	91<br>82	-	36<br>37	43<br>35	59<br>35	29<br>27	-	96<br>77
L. kmetyi	C<br>I	95<br>43	99<br>49	99<br>49	95<br>44.20	9<br>73	96<br>47	96<br>48	98<br>48	98<br>48	100<br>35	94<br>44	98<br>50	64<br>45	100<br>78	91<br>41	36<br>38	95<br>39	94<br>40	40<br>27	-	96<br>78
L. tipperaryensis	C<br>I	74<br>32	99<br>52	99<br>51	86<br>44	9<br>64	95<br>47	96<br>49	96<br>49	94<br>49	87<br>37	89<br>49	89<br>48	61<br>49	100<br>72	96<br>40	36<br>37	95<br>44	75<br>49	29<br>25	-	94<br>75
L. putramalaysiae/ L. stimsonii	C<br>I	99<br>43	100<br>43	100<br>43	97<br>40	98<br>37	96<br>45	100<br>43	100<br>38	100<br>38	88<br>43	99<br>36	98<br>46	93<br>33	92<br>74	95<br>40	16<br>48	96<br>43	87<br>44	48<br>28	-	90<br>76
L. adleri	C<br>I	86<br>51	100<br>37	100<br>37	86<br>50	37<br>29	89<br>48	91<br>54	97<br>36	96<br>54	90<br>34	98<br>36	97<br>51	65<br>48	99<br>73	77<br>43	36<br>35	91<br>45	77<br>48	29<br>27	-	97<br>74
L. ellisii	C<br>I	45<br>29	98<br>32	49<br>35	40<br>39	9<br>70	54<br>34	78<br>34	46<br>33	33<br>33	29<br>32	18<br>36	60<br>34	67<br>30	89<br>76	-	36<br>36	-	77<br>33	4<br>66	-	92<br>70
L. gomenensis	C<br>I	87<br>52	99<br>49	99<br>49	100<br>46	9<br>66	89<br>41	90<br>35	93<br>35	83<br>43	90<br>48	99<br>48	98<br>50	79<br>38	97<br>69	79<br>40	36<br>33	97<br>46	68<br>49	5<br>55	-	98<br>67
L. licerasiae	C<br>I	44<br>29	51<br>30	49<br>29	-	-	45<br>30	34<br>28	-	-	-	35<br>27	-	24<br>33	98<br>40	-	-	-	32<br>35	-	-	93<br>34
L. hartskeerlii	C<br>I	44<br>29	50<br>29	47<br>29	-	-	45<br>30	34<br>29	-	-	-	35<br>27	-	27<br>32	98<br>38	-	-	-	32<br>34	-	-	93<br>34
L. dzoumogneensis	C<br>I	44<br>29	50<br>31	47<br>29	-	-	45<br>30	34<br>27	-	-	-	35<br>29	-	24<br>33	97<br>39	-	-	-	32<br>35	-	-	93<br>34
L.venezuelensis	C<br>I	44<br>28	77<br>31	47<br>28	-	-	45<br>29	34<br>28	-	-	-	35<br>27	80<br>33	24<br>31	98<br>40	-	-	-	45<br>31	-	-	93<br>33
L. selangorensis	C<br>I	44<br>29	51<br>30	87<br>44	79<br>34	-	45<br>29	88<br>42	79<br>44	61<br>37	51<br>34	35<br>28	89<br>50	57<br>45	98<br>41	-	-	56<br>39	54<br>45	7<br>36	-	84<br>36
L. haakeii	C<br>I	44<br>29	50<br>31	49<br>28	-	-	45<br>30	34<br>28	-	-	-	35<br>27	-	24<br>33	98<br>40	-	-	-	32<br>35	-	-	93<br>35
L. andrefontaineae	C<br>I	44<br>29	76<br>31	49<br>28	-	-	45<br>30	34<br>28	-	-	-	35<br>27	80<br>33	24<br>33	98<br>40	-	-	-	32<br>35	-	-	93<br>33

Table S1: Amino acid sequence identities of LRRs from *Leptospira* spp. Similarity is shown in yellow above 70%, in blue between 40% and 70% and in green below 40%

Proteins	%	LIC10828	LIC10829	LIC10830	LIC10831	LIC11051	LIC11097	LIC11098	LIC11180	LIC11504	LIC11507	LIC11505	LIC12234	LIC12375	LIC12401	LIC12512	LIC12676	LIC12759	LIC12899	LIC12901	LIC20055	LIC20154
L. koniambonensis	C<br>I	44<br>29	70<br>36	49<br>29	-	30<br>33	45<br>31	34<br>29	72<br>34	-	-	35<br>29	48<br>35	24<br>33	98<br>40	-	-	-	45<br>31	10<br>36	100<br>43	93<br>36
L. neocaledonica	C<br>I	44<br>29	51<br>31	43<br>31	-	-	45<br>32	34<br>29	-	-	-	35<br>28	-	24<br>33	98<br>40	-	-	-	32<br>35	-	100<br>41	96<br>34
L. johnsonii	C<br>I	44<br>28	51<br>29	49<br>28	-	-	45<br>32	34<br>27	-	-	-	35<br>27	-	24<br>31	98<br>40	-	-	-	32<br>35	-	-	93<br>34
L. sarikeiensis	C<br>I	44<br>29	50<br>32	43<br>32	-	-	45<br>29	34<br>29	-	-	-	35<br>27	-	24<br>34	98<br>42	-	-	-	32<br>36	-	-	84<br>36
L. langatensis	C<br>I	44<br>26	47<br>29	-	-	-	-	29<br>30	-	-	-	9<br>35	80<br>33	19<br>33	99<br>38	-	-	-	45<br>31	-	-	-
L. semungkisensis	C<br>I	44<br>26	46<br>28	-	-	-	-	-	-	-	-	-	-	19<br>33	97<br>38	-	-	-	30<br>33	-	-	27<br>31
L. wolffi	C<br>I	-	-	-	-	-	-	-	-	-	-	-	-	-	92<br>43	-	-	-	-	-	-	83<br>35
L. fletcheri	C<br>I	-	-	-	-	18<br>38	-	34<br>27	-	-	-	-	-	-	91<br>45	76<br>34	-	-	-	-	-	93<br>35
L. fluminis	C<br>I	-	-	-	-	-	-	34<br>27	-	-	-	-	-	-	95<br>43	85<br>33	-	-	-	-	-	85<br>36
L. broomii	C<br>I	-	-	26<br>29	-	-	-	-	-	-	-	-	-	-	91<br>44	-	19<br>33	33<br>32	-	-	-	93<br>34
L. inadai	C<br>I	42<br>35	24<br>35	26<br>31	-	-	-	-	-	-	-	42<br>33	-	-	91<br>42	-	-	31<br>33	21<br>33	-	-	93<br>35
L. fainei	C<br>I	44<br>32	26<br>32	28<br>33	-	-	46<br>31	-	50<br>31	-	-	35<br>28	-	-	91<br>43	-	-	-	-	-	-	84<br>34
L. perolatii	C<br>I	-	26<br>32	-	36<br>34	23<br>32	33<br>34	56<br>31	77<br>35	-	-	-	79<br>34	24<br>32	96<br>42	-	-	56<br>30	-	9<br>34	-	97<br>37
L. biflexa	C<br>I	80<br>38	98<br>38	96<br>40	81<br>41	-	93<br>37	95<br>34	94<br>36	71<br>30	83<br>35	79<br>34	80<br>44	-	95<br>40	-	-	87<br>39	76<br>34	6<br>37	-	-
L. bouyouniensis	C<br>I	82<br>38	98<br>38	87<br>41	87<br>39	-	94<br>32	95<br>39	91<br>39	71<br>35	71<br>36	81<br>36	93<br>43	60<br>43	94<br>38	-	-	72<br>41	80<br>36	-	-	-
L. jelokensis	C<br>I	82<br>38	94<br>38	91<br>35	86<br>39	-	92<br>33	90<br>35	90<br>39	68<br>33	81<br>33	71<br>33	93<br>43	64<br>40	89<br>39	-	-	79<br>41	80<br>37	13<br>30	-	-
L. yanagawae	C<br>I	82<br>40	98<br>40	91<br>43	87<br>40	-	91<br>42	96<br>35	90<br>38	62<br>33	87<br>36	81<br>37	93<br>44	86<br>42	94<br>38	45<br>43	-	98<br>39	80<br>38	13<br>33	-	-
L. kemamanensis	C<br>I	80<br>40	92<br>40	96<br>42	87<br>44	-	90<br>37	95<br>35	94<br>34	71<br>35	86<br>36	79<br>34	77<br>45	71<br>35	95<br>40	41<br>45	-	95<br>39	93<br>37	13<br>32	-	-
L. levettii	C<br>I	80<br>39	98<br>39	89<br>41	87<br>42	-	87<br>35	95<br>33	95<br>33	65<br>36	87<br>36	68<br>37	89<br>44	71<br>35	93<br>39	41<br>47	-	87<br>39	74<br>36	13<br>29	-	-
L. ellinghausenii	C<br>I	80<br>38	98<br>38	86<br>39	95<br>39	-	93<br>36	91<br>32	94<br>33	65<br>36	72<br>36	68<br>37	89<br>42	61<br>41	94<br>38	-	-	95<br>39	74<br>37	11<br>30	-	-
L. brenneri	C<br>I	80<br>39	95<br>39	91<br>43	95<br>37	-	94<br>40	96<br>42	72<br>38	85<br>33	75<br>30	87<br>34	93<br>37	77<br>34	95<br>38	-	-	81<br>37	93<br>35	6<br>33	-	-
L. perdikensis	C<br>I	80<br>38	98<br>37	96<br>43	90<br>37	-	88<br>35	89<br>33	87<br>33	86<br>33	91<br>29	90<br>38	83<br>43	90<br>37	95<br>36	41<br>45	-	95<br>38	93<br>36	6<br>39	-	-
L. terpstrae	C<br>I	80<br>36	99<br>42	96<br>43	91<br>35	-	95<br>35	96<br>44	81<br>40	89<br>31	99<br>34	86<br>37	93<br>39	77<br>34	95<br>37	41<br>49	-	97<br>39	74<br>36	6<br>38	-	-
L. vanthielii	C<br>I	55<br>36	99<br>36	96<br>39	78<br>39	-	87<br>34	94<br>34	87<br>35	89<br>32	87<br>42	86<br>37	83<br>41	90<br>37	95<br>34	41<br>45	-	95<br>37	93<br>34	6<br>39	-	-

Table S1: Amino acid sequence identities of LRRs from *Leptospira* spp. Similarity is shown in yellow above 70%, in blue between 40% and 70% and in green below 40%

Proteins	%	LIC10828	LIC10829	LIC10830	LIC10831	LIC11051	LIC11097	LIC11098	LIC11180	LIC11504	LIC11507	LIC11505	LIC12234	LIC12375	LIC12401	LIC12512	LIC12676	LIC12759	LIC12899	LIC12901	LIC20055	LIC20154
L. congkakensis	C<br>I	90<br>39	95<br>39	96<br>41	83<br>42	-	87<br>42	86<br>46	91<br>37	62<br>32	91<br>30	81<br>36	83<br>39	63<br>38	95<br>37	44<br>42	-	87<br>37	80<br>34	-	-	-
L. noumeaensis	C<br>I	90<br>39	95<br>39	96<br>41	82<br>42	-	87<br>41	86<br>44	91<br>39	71<br>32	91<br>30	81<br>36	83<br>39	90<br>37	95<br>36	44<br>44	-	87<br>38	80<br>34	-	-	-
L. kanakyensis	C<br>I	90<br>39	98<br>39	96<br>41	82<br>42	-	87<br>40	94<br>44	91<br>39	62<br>33	91<br>34	81<br>37	80<br>38	90<br>37	95<br>37	44<br>44	-	87<br>38	80<br>34	-	-	-
L. mtsangambouensis	C<br>I	67<br>38	95<br>38	91<br>41	90<br>37	-	95<br>35	95<br>43	87<br>33	65<br>33	85<br>34	86<br>37	89<br>42	68<br>44	95<br>37	41<br>47	-	87<br>36	93<br>36	-	-	-
L. bourretii	C<br>I	55<br>37	95<br>41	96<br>40	90<br>37	-	95<br>35	95<br>43	87<br>31	65<br>32	85<br>35	86<br>36	83<br>43	68<br>43	95<br>38	41<br>47	-	95<br>36	93<br>35	-	-	-
L. meyeri	C<br>I	82<br>39	94<br>39	95<br>42	82<br>42	-	88<br>35	94<br>45	91<br>40	71<br>33	87<br>32	81<br>38	80<br>39	63<br>38	95<br>38	44<br>46	-	87<br>38	93<br>37	6<br>32	-	-
L. harrisiae	C<br>I	57<br>39	95<br>39	96<br>41	59<br>34	-	91<br>38	89<br>43	74<br>40	65<br>32	76<br>33	83<br>35	83<br>42	68<br>35	86<br>41	41<br>49	-	80<br>35	81<br>34	-	-	-
L. bandrabouensis	C<br>I	80<br>40	92<br>41	90<br>41	90<br>38	-	95<br>34	90<br>43	64<br>38	65<br>32	72<br>35	86<br>35	89<br>44	72<br>42	95<br>38	41<br>49	-	92<br>35	74<br>35	-	-	-
L. montravelensis	C<br>I	80<br>38	99<br>45	96<br>41	97<br>37	-	92<br>35	84<br>44	87<br>31	74<br>36	72<br>35	86<br>37	83<br>43	71<br>36	86<br>40	41<br>49	-	82<br>37	93<br>35	-	-	-
L. idoni	C<br>I	85<br>46	98<br>41	88<br>40	85<br>45	52<br>33	92<br>41	88<br>42	89<br>41	73<br>43	91<br>36	89<br>38	97<br>45	62<br>41	89<br>39	-	-	86<br>40	81<br>34	23<br>30	-	-
L. ilyithenensis	C<br>I	-	25<br>30	-	-	11<br>35	23<br>36	86<br>35	53<br>29	-	-	44<br>30	37<br>38	44<br>29	-	-	-	-	72<br>35	8<br>31	-	-
L. kobayashii	C<br>I	38<br>30	57<br>32	-	-	11<br>39	41<br>36	86<br>30	53<br>29	-	20<br>33	47<br>31	38<br>39	44<br>28	94<br>34	-	-	34<br>29	59<br>34	8<br>31	-	-
L. ognonensis	C<br>I	43<br>35	39<br>35	72<br>38	62<br>32	-	40<br>36	73<br>33	55<br>38	49<br>30	28<br>43	39<br>31	75<br>39	-	99<br>36	-	-	37<br>37	48<br>44	5<br>36	-	-
L. ryugenii	C<br>I	73<br>31	95<br>36	91<br>32	31<br>32	-	87<br>33	84<br>35	78<br>33	25<br>37	57<br>33	63<br>31	89<br>33	57<br>34	88<br>36	42<br>38	-	76<br>34	76<br>32	-	-	-

Table S1: Amino acid sequence identities of LRRs from *Leptospira* spp.
