# Supplementary material for: New insights into the putative role of leucine-rich repeat proteins of Leptospira interrogans and their participation in host cell invasion: an in silico analysis
Source: Front Cell Infect Microbiol. 2024 Dec 13;14:1492352. doi: 10.3389/fcimb.2024.1492352 (PMC11674859; doi:10.3389/fcimb.2024.1492352)
Supplement: Supplementary file 2 [file DataSheet2.pdf]

S2 table: Prediction of molecular, biological and cellular function by GO

| Molecular Function | | | Biological Process | | | Cellular component | | |
|---|---|---|---|---|---|---|---|---|
| **ID** | **Name** | **Hits** | **ID** | **Name** | **Hits** | **ID** | **Name** | **Hits** |
| GO:0005525 | GPT Binding | 17 | GO:0007264 | small GTPase mediated signal transduction | 17 | GO:0005622 | Intracellular | 16 |
| GO:0004016 | adenylate cyclase activity | 15 | GO:0006171 | cAMP biosynthetic process | 7 | GO:0016020 | membrane | 6 |
| GO:0004842 | ubiquitin-protein transferase activity | 14 | GO:0016567 | protein ubiquitination | 9 | GO:0005634 | nucleus | 2 |
| GO:0004672 | protein kinase activity | 7 | GO:0007165 | signal transduction | 9 | GO:0005654 | nucleoplasm | 1 |
| GO:0016829 | lyase activity | 2 | GO:0006468 | protein phosphorylation | 8 | GO:0030054 | cell junction | 1 |
| GO:0004721 | phosphoprotein phosphatase activity | 1 | GO:0006266 | DNA ligation | 1 | GO:0016021 | integral component of membrane | 5 |
| GO:0098641 | cadherin binding involved in cell-cell adhesion | 1 | GO:0006310 | DNA recombination | 1 | GO:0005737 | cytoplasm | 1 |
| GO:0003910 | DNA ligase (ATP) activity | 1 | GO:0006281 | DNA repair | 1 | GO:0034750 | Scrib-APC-beta-catenin complex | 1 |
| GO:0016874 | ligase activity | 1 | | | | GO:0035748 | myelin sheath abaxonal region | 1 |
| GO:0005524 | ATP binding | 1 | | | | | | |

**Virulence factors of other pathogens**

*Listeria monocytogenes* InlB

| Molecular Function | | | Biological Process | | | Cellular component | | |
|---|---|---|---|---|---|---|---|---|
| **ID** | **Name** | | **ID** | **Name** | | **ID** | **Name** | |
| GO:0005515 | protein binding | | GO:0007264 | small GTPase mediated signal transduction | | GO:0016021 | integral component of membrane | |
| GO:0005525 | GTP binding | | | | | GO:0016020 | membrane | |

*Yersinia pestis* YopM

| Molecular Function | | | Biological Process | | | Cellular component | | |
|---|---|---|---|---|---|---|---|---|

| ID | Name | | ID | Name | | ID | Name | |
|---|---|---|---|---|---|---|---|---|
| GO:0004842 | ubiquitin-protein transferase activity | | GO:0016567 | protein ubiquitination | | GO:0005576 | extracellular region | |
| GO:0046872 | metal ion binding | | | | | GO:0009279 | cell outer membrane | |
| GO:0016874 | igase activity | | | | | GO:0016020 | membrane | |
| | | | | *Streptococcus pyogenes* SLR | | | | |
| | Molecular Function | | | Biological Process | | | Cellular component | |
| ID | Name | | ID | Name | | ID | Name | |
| GO:0005525 | GTP binding | | GO:0007264 | small GTPase mediated signal transduction | | GO:0016021 | integral component of membrane | |
| | | | | | | GO:0016020 | membrane | |
| | | | | *Streptococcus agalactiae* LRRG | | | | |
| | Molecular Function | | | Biological Process | | | Cellular component | |
| ID | Name | | ID | Name | | ID | Name | |
| GO:0010181 | FMN binding | | GO:0000272 | polysaccharide catabolic process | | GO:0016020 | membrane | |
| GO:0005509 | calcium ion binding | | | | | GO:0016021 | integral component of membrane | |
| | | | | *Salmonella enterica* SLRP | | | | |
| | Molecular Function | | | Biological Process | | | Cellular component | |
| ID | Name | | ID | Name | | ID | Name | |
| GO:0004842 | ubiquitin-protein transferase activity | | GO:0016567 | protein ubiquitination | | GO:0005576 | extracellular region | |
| GO:0016874 | ligase activity | | | | | GO:0030430 | host cell cytoplasm | |
| | | | | | | GO:0042025 | host cell nucleus | |
| | | | | *Treponema denticola* LRRA | | | | |
| | Molecular Function | | | Biological Process | | | Cellular component | |
| ID | Name | | ID | Name | | ID | Name | |

| | | | | | | | | |
|---|---|---|---|---|---|---|---|---|
| GO:0005509 | calcium ion binding | | GO:0000272 | polysaccharide catabolic process | | GO:0005615 | extracellular space | |
| GO:0004842 | ubiquitin-protein transferase activity | | GO:0016567 | protein ubiquitination | | GO:0016021 | integral component of membrane | |
| GO:0004553 | hydrolase activity, hydrolyzing O-glycosyl compounds | | | | | GO:0016020 | membrane | |
| GO:0030246 | carbohydrate binding | | | | | | | |
| *Treponema pallidum* TpLRR | | | | | | | | |
| Molecular Function | | | Biological Process | | | Cellular component | | |
| **ID** | **Name** | | **ID** | **Name** | | **ID** | **Name** | |
| GO:0005509 | calcium ion binding | | GO:0000272 | polysaccharide catabolic process | | GO:0005615 | extracellular space | |
| GO:0004553 | hydrolase activity, hydrolyzing O-glycosyl compounds | | | | | GO:0016020 | membrane | |
| | | | | | | GO:0016021 | integral component of membrane | |
